# Applications and Therapeutic Actions of Complementary and Alternative Medicine for Women with Genital Infection

**DOI:** 10.1155/2014/658624

**Published:** 2014-02-04

**Authors:** Chenfang Liu, Yuehui Zhang, Sai Kong, Ilene Tsui, Yang Yu, Fengjuan Han

**Affiliations:** ^1^Department of Obstetrics and Gynecology, Heilongjiang University of Chinese Medicine, Harbin, Heilongjiang 150040, China; ^2^Department of Obstetrics and Gynecology, First Affiliated Hospital, Heilongjiang University of Chinese Medicine, Harbin, Heilongjiang 150040, China; ^3^Center for Post-Doctoral Studies, Heilongjiang University of Chinese Medicine, Harbin 150040, China; ^4^Pennsylvania State University College of Medicine, Hershey, PA 17033, USA

## Abstract

Genital infection is a common worldwide disease among females with clinical features such as bilateral lower abdominal tenderness, abnormal vaginal or cervical discharge, fever, abnormal vaginal bleeding, dyspareunia, vaginal itching, and adnexal tenderness, which can significantly impair women's health and quality of life. Genital infection is commonly treated with antibiotics, leading to an imbalance in gut flora due to prolonged use of antibiotics. Therefore, it is necessary to discover safe and efficacious alternative treatment strategies for patients with genital infection. Complementary and alternative medicine (CAM) is becoming increasingly prevalent among women with genital infection. CAM has interested the western mainstream medical community because of its less invasive, safe, effective, economical, and convenient therapies. CAM focuses on the prevention and treatment of disease and has become an important force in treating chronic disease. During the last few decades, the popularity of CAM has gradually increased. To further understand the efficacy of CAM in treating genital infection, our paper will review the current progress of treating genital infection including vulvitis, vaginitis, cervicitis, and pelvic inflammatory disease (PID) with CAM therapies. Several CAM strategies including traditional Chinese medicine (TCM), acupuncture, Psychology interference, and physical therapy are introduced in this review.

## 1. Introduction

Genital infection is common among women of all ages and includes a variety of different diseases of the genital tract including vulvitis, vaginitis, cervicitis, and pelvic inflammatory disease (PID). Infection plays an important role in gynecology and infertility, affecting the ovary, uterus; and the embryo and implantation [1]. Long-term and repeated infections will cause damage and adhesion of tubal mucosa, eventually leading to infertility. Due to the nature of these wide-ranging effects, it is important to find effective therapies for genital infection. Currently, genital infection is commonly treated with antibiotics. As a side effect, the increased drug-resistance has reduced therapeutic efficacy. CAM is defined as the complement for conventional medical therapy, providing diagnosis, therapy, and prevention that conventional medical regimens otherwise cannot treat. CAM mainly contains the following methods: (i) alternative medical system: TCM, Ayurveda, and homeopathy; (ii) mind-body intervention: meditation and biofeedback; (iii) biologically based therapies: herbal therapy and special diet therapy; (iv) manipulative and body-based methods: chiropractic and massage; (v) energy therapies [2]. At present, several CAM methods have been used in treating genital infection. This review briefly summarizes the current progress of treating genital infection with CAM and introduces the potential mechanisms.

## 2. TCM Utilization in Genital Infection

As a form of primary care throughout many Asian countries, TCM is an important part of CAM. The basic TCM therapies include Chinese herbal medicine (CHM) and acupuncture. Chinese herbs and acupuncture are important components of TCM. They have been used for disease treatment and prevention and as alternative therapies for over 2000 years [3]. TCM has significant advantages in treating genital infection. In TCM, genital infection is usually classified as the area of “leukorrheal diseases,” “the woman abdominal pain,” and “abdominal mass” and the treatment is commonly based on syndrome differentiation. CHM acts on “Zang-fu” viscera internally and part of skin externally, while acupuncture works by stimulating certain areas of the external body. Indeed, a number of articles published within the last decade have examined the use of TCM for the treatment of genital infection.

### 2.1. The Effect of Chinese Herbs on Genital Infection

Based on TCM theory of syndrome differentiation, genital infection is divided into several TCM syndrome types, and then different traditional Chinese medicines are composed together to treat different syndrome types. Two approaches can be used in treatment: oral and external approaches, which can be applied independently or in combination. According to the theory of TCM, the pathogen of genital infection can be summarized as follows: heat, toxin, damp, stasis, and cold. The accumulation of these factors in the genitals leads to pathological changes, causing the symptoms of genital infection.

#### 2.1.1. Oral Administration of CHM for Genital Infection in TCM

Clinical research has shown that Chinese medicinal compounds have certain satisfactory effects on genital infection including vulvitis, vaginitis, cervicitis, and PID. These results were used to evaluate whether Chinese herbs could be used as a complementary drug to treat specific symptoms. Among all the formulae studied for various genital inflammatory diseases, “Shaofuzhuyutang” et al. (Table 1) were the most frequently used formulas and demonstrated a higher effect on reducing genital infectious diseases.

#### 2.1.2. External Therapy to Genital Infection in TCM

External therapy of TCM is a form of traditional medicine therapy, preventing and curing diseases by stimulating the meridians, acupuncture points, skin, mucous membranes, muscles, and bones. External therapy has experienced a long history, dating back to ancient China, and gradually developed into a unique medical approach. There are two main approaches in external therapy. (1) Skin penetration and mucosal absorption: there are many ways of transdermal administration with Chinese medicine, such as dressing, stickers, steaming, washing, and bathing. Most drugs are easily absorbed from the dermis to the human body after passing through the skin. The greatest advantage of transdermal administration is that it avoids potentially damaging the gastrointestinal tract and liver while also avoiding metabolic effects of these respective organs, thereby increasing drug effect and maintaining plasma concentration over a sustained period of time [33]. (2) Mucosal absorption: the route of administration is through the mucosa, including mouth, eyes, nose, genitalia, and anus. We have reviewed several related studies that verify the efficacy of CHM as external therapy for various genital infectious diseases (Table 2).

*(i) Rectal Administration*. Currently, rectal administration includes retention enema and rectal infusion with liquid Chinese herbal medicine [52]. Chronic pelvic inflammatory disease (CPID) mainly spreads inside the pelvic cavity, while the rectum neighbors both the uterus and uterine adnexa, making rectal administration a plausible method of drug delivery. There are dense veins in female pelvic tissues and organs with rich blood supply. Since the rectal mucosa is relatively thin, the medicine will directly act on the pelvic cavity at a high concentration. Liquid Chinese herbal medicine by rectal administration was shown to be a good treatment for CPID. This method not only promotes blood circulation, improves tissue nutrition, and reduces the inflammatory exudate but also inhibits the proliferation of connective tissues, thereby promoting the absorption of the inflammatory mass, releasing tissue adhesions and relieving local spasms [53]. Further, retention enema also avoids the hepatic first-pass effect, reducing the burden on the liver.

*(ii) Steaming Washing Therapy*. Steaming washing therapy is a kind of traditional Chinese medicine therapy, where patients are usually told to take a hip bath with hot liquid herbal medicine. Before washing the certain area with the liquid, the steam can be absorbed to a certain degree. Both Chinese herbal compounds and the heat coordinate to accelerate the absorption of the medicine into the certain location [54].

*(iii) Vaginal Lavage*. Vaginal lavage is commonly used to treat vaginitis and cervicitis with various functions of cleaning the vagina, promoting vaginal blood circulation, reducing vaginal secretions, and relieving local hyperemia. Vaginal lavage with Chinese medicine serves two purposes: (1) to change the pH value within the vagina and inhibit or kill the *Trichomonas* bacteria; (2) to remove a large amount of vaginal secretions, thereby greatly reducing the number of pathogenic microorganisms. The clinical observation has shown satisfactory efficacy from the treatment of *Trichomonas vaginitis* by vaginal lavage with traditional Chinese medicine [55].

*(iv) Intravaginal Administration*. Some clinical observations reported that traditional Chinese medicine had achieved good effects on PID and vaginitis in treatment with the intravaginal-drug way. One study showed that the total effective rate was 98.2% treating PID in the vaginal fornix with the paste composed of Shuanghuanglian powder 0.6 g, Huoxuezhitong scattered 3 g, and Tetramethylpyrazine 2 mL [56].

*(v) External Application*. Grind the CHM into fine powder and put it in a warm bag. Applying the bag externally to the lower abdomen increases the drug concentration at certain focal points and allows direct absorption by heat conduction. External application helps to ameliorate blood circulation in the pelvic cavity and promote the absorption of inflammation [57]. Further, this method to a certain extent is able to avoid stimulating the gastric mucosa, so that the bioavailability of the drug can be prolonged.

#### 2.1.3. Single Herbs and Phytotherapy

*Single Herbs*. Chinese medicine has various functions including heat-clearance, blood-activation, and stasis-elimination and acts as a general anti-inflammatory or analgesic and improves the immune function by focusing on the several pathogenic characteristics of the gynecological inflammation. *Smilax* is mainly used for the treatment of CPID and gynecological inflammation in clinic. An experimental study has shown that *Smilax* can inhibit the hyperplasia of endometrial inflammatory cells, promote the recovery of damaged epithelial cells, and reduce congestion and edema of serosa for CPID in rat models [58]. A pharmacodynamic screening found that the ethyl acetate extracted from *Smilax* was the effective fraction for CPID treatment with an anti-inflammatory function. The anti-inflammatory substances of *Smilax* were ingredients of flavonoids, saponins, and tannins [59]. Comfrey oil, extracted from comfrey, which is Radix Lithospermi Root of Sinkiang Arnebia (a kind of Chinese herb medicine), was commonly used to treat candida vaginitis with a higher efficacy rate, lower recurrence, and minimal side effects. The effective ingredient of comfrey oil is Shikonin C_16_H_16_O_5_; it could be absorbed rapidly in the vagina, then alleviating or curing the symptoms after treating for 3 to 4 days, and the cure rate is 92% [60]. Wild chrysanthemum suppository, extracted from the wild chrysanthemum, was proved to have a good effect on CPID by rectal administration with more rapid, sustained effects and less adverse effects [61]. *Patrinia* is a kind of perennial herb in Valerianaceae, containing a variety of saponins which inhibit a variety of bacterial and virus. *Patrinia* is mainly used to treat CPID due to a strong inhibitory effect on *Staphylococcus aureus*, *Bacillus anthracis*, *Bacillus diphtheria*, hepatitis B *Streptococcus*, *Salmonella typhi*, and *Shigella* [62]. *Houttuynia cordata*, drying part of the perennial herb *Houttuynia* (a kind of Chinese herb medicine), containing complex composition such as volatile oils, alkaloids, flavonoids, and polysaccharide which can inhibit a variety of bacteria and relieve pain and inflammation, has evidenced a good efficacy for CPID [63, 64]. Some related studies have verified that some single herbs such as Cortex Moutan Radicis, Rhizoma Curcumae, and Radix Paeoniae can promote blood circulation and the inflammatory exudate absorption. In summary, single herb is an effective therapy for CPID and further deep clinical studies should be done in the near future. We have made a list of some frequent single herbs in treating various genital inflammatory diseases (Table 3), and they are also quite common in Chinese herbal compounds for genital infectious diseases.

*Phytotherapy*. Significant research has been done to evaluate the efficacy of some plants and their active extract against vaginal pathogens and has demonstrated that they could provide an effective approach for treatment of vaginitis [149]. Phytotherapy is mainly used to treat vaginitis with herbal medicines which are anticandida, antibacterial, and anti-Trichomonas. These active extracts including carvacrol, 1,8-cineole, geranial, germacrene-D, limonene, linalool, menthol, terpinen-4-ol, and thymol exhibit the beneficial effects on many types of vaginitis including bacterial vaginosis, vulvovaginal candidosis, and *Trichomonas vaginitis*. These extracts can block mycelial growth at a very low concentration according to human and animal studies [150] and may present a new direction for the future role of plants in treating vaginitis.

### 2.2. Mechanisms of Chinese Herbal Medicine in Treating Genital Infection

**Figure 1:**
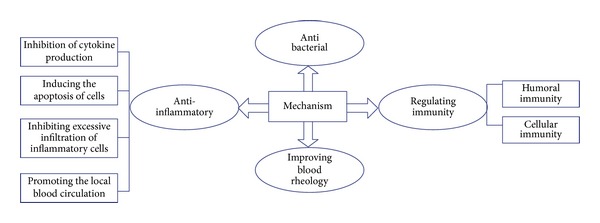
Mechanisms of Chinese herbal medicine on genital infection.

Modern medicine indicates that genital infection is mainly caused by the presence of pathogenic microorganisms, a declining immune system, or a pelvic microcirculation disorder [3]. Therefore, medication treatment is mainly focused on targeting pathogenic microorganisms, improving immunity, or promoting blood circulation. In recent years, some experimental studies have focused on the mechanism of treating genital infection with Chinese herbal compounds and Chinese herbal patents (Figure 1).

#### 2.2.1. Antibacterial Mechanism of Chinese Herbal Medicine

In many cases, microorganisms from the vaginal and cervical flora are frequently associated with PID, including anaerobic and facultative bacteria, similar to those associated with bacterial vaginosis [151]. The laboratory study confirms that Chinese medicine has an inhibitory effect on certain pathogens. Ziying granule, which consists of Tokyo Violet Herb, Dandelion, and 7 other herbs, has a broad bactericidal effect on common pathogenic bacteria found in the pelvic cavity [152]. Penyanqing Granule, which consists of Amur Cork-Tree Bark, *Paeonia suffruticosa*, and several other herbs, has also been shown to be an effective Chinese herbal compound for the clinical treatment of PID. A study result has shown that the common pathogens of *Escherichia coli* and *Staphylococcus aureus* were inhibited by Penyanqing Granule in PID mice model and have an obvious dose-dependent effect [153]. Furthermore an experiment on the effect of different doses of *Smilax* on common gynecological bacterial infections has shown that it had the broadest antibacterial spectrum at the concentration of 2 g/mL, inhibiting *S. aureus*, *E. coli*, *P. aeruginosa*, and *P. mirabilis* significantly and regulating the vaginal pH value [154, 155]. All of these therapies apply a promising alternative for treatment of genital infection.

#### 2.2.2. Anti-Inflammatory Mechanism of Chinese Herbal Medicine

Inflammation is a defense reaction between the body and various damaging factors. The inflammatory response depends on the nature and intensity of the pathogenic factor and the reactivity of the body [151]. The inflammatory response is primarily activated through a series of inflammatory mediators. The anti-inflammatory mechanism of certain Chinese herbs can alleviate secondary tissue injury and eliminate or reduce inflammation by inhibiting the synthesis of inflammatory mediators, controlling the infiltration of inflammatory cells appropriately, and inducing apoptosis of infiltrated inflammatory cells.

*(i) Inhibition of Cytokine Production*. Chinese medicine may exhibit an anti-inflammatory effect by inhibiting the production of the cytokine secreted by activating lymphocytes, plasma cells, macrophages, and monocytes, such as IL-4 (interleukin-4) and TNF-*α* (tumor necrosis factor-*α*). TNF-*α* is one of the first appearing inflammatory factors in the inflammatory response, which has a strong proinflammatory role in the early inflammatory response [156].

The clinical study showed that “Penqiangyan granule,” which consisted of *Salvia miltiorrhiza*, Radix Paeoniae Rubra, *Cuscuta chinensis*, Pollen Typhae, and so forth (some kinds of Chinese herbal medicine), can significantly decrease the serum concentration of TNF-*α* with CPID patients to normal level. Furthermore, the experimental study found that the serum TNF-*α* concentration was higher in CPID model rat than the normal rat, while the serum TNF-*α* level was significantly decreased after treatment with “Penqiangyan granule” and reached normal level, which implied that the possible mechanism of “Penqiangyan granule” treatment on CPID was improving immune condition and inhibiting inflammatory cytokines [157]. Yan Ting, a kind of enemas of Chinese herbal medicine, consisting of Radix Paeoniae Rubra, *Corydalis yanhusuo*, *Salvia miltiorrhiza*, *Sparganium stoloniferum*, Rhizoma Curcumae, Sargentgloryvine Stem, and so forth, has proved that it could decrease the mRNA expression of TNF-*α* in fallopian tube, ease the adhesions and promote the recanalization of fallopian tube, and eventually improve the pregnancy rates in the rat model of salpingitis infertility [158].

*(ii) Inducing the Apoptosis of Cells and Inhibiting Excessive Infiltration of Inflammatory Cells*. CHM can induce cellular apoptosis, thereby reducing cell necrosis, effectively removing the inflammatory lesions of inflammatory cells and other proliferating cells, reducing secondary injury due to adjacent tissue cell necrosis, preventing inflammatory fibrous hyperplasia and scar. Furthermore, CHM can inhibit excessive infiltration of inflammatory cells by inhibiting hyperplasia of fibroblast and increasing degradation of fibrous tissue [159]. The activated nuclear factor kB (NF-KB) can inhibit or delay the apoptosis of polymorphonuclear (PMN), producing large amounts of cytokines which accelerated local and systemic inflammatory response. Caspase-3 is a key enzyme in the downstream of apoptotic pathway; caspase-3 activated will promote apoptosis factors, ultimately leading to apoptosis through caspase-3-mediated signaling pathways, while CHM can induce apoptosis by regulating the expression of NF-KB and caspase-3 to reduce the inflammatory response [160, 161]. Study results suggested that the protein level of caspase-3 was increased in the endometrium of CPID rat model with “Penyankang (a kind of compound of CHM consists (sic) of Caulis Sargentodoxae, Herba Patriniae, Salvia Miltiorrhiza and Sparganium Stoloniferum)” treatment than control group, which led to an increase of cell apoptosis, thereby reducing the inflammatory response [159]. Matrix metalloproteinase enzymes (MMP) play a major role in the process of extracellular matrix degradation, which can degrade almost all components of the extracellular matrix. The expression of MMP in the tissue of normal state is minimal, while it increases after the stimulation by inflammatory cytokines, hormones, and growth factors and also increases in the process of cell transformation [162]. The experimental results showed that the protein level of MMP had been enhanced after treatment with “Penyankang” in CPID rat model, which led to a degradation of the extracellular matrix, thereby reducing proliferation of the fibrous tissue in the chronic inflammation [160].

#### 2.2.3. The Fever-Reducing and Analgesic Effect of Chinese Herbal Medicine

The great majority of women with PID are suffering from chronic pain, which can significantly influence their activities of daily life. Chronic pain management is an increasing challenge to the treatment for PID patients [163]. In recent years, many agents were commonly used to control chronic pain, such as nonsteroidal anti-inflammatory drugs (NSAIDs) and long-acting opiates, but NSAIDs have the hidden danger of serious toxicity, including gastrointestinal bleeding and renal failure, while the long-term efficacy of long-acting opiates has been poorly documented, and there is a significant potential for addiction and abuse [164–166]. Obviously, new approaches are necessary for managing chronic pain, especially low-risk interventions with the potential to reduce the persistent pain.

*Smilax* is the rhizome of *Smilax china L*, belonging to Liliaceae plant. The preparations based on *Smilax* are widely used to treat CPID and other genial inflammations. A study found that the “Smilax capsules” can significantly inhibit the writhing response compared with the control group in PID mice model induced by acetic acid. Both of the higher and lower doses could significantly reduce mice writhing within 20 minutes, exhibiting obvious analgesic and anti-inflammatory effects [167, 168].

#### 2.2.4. The Mechanism of Chinese Herbal Medicine in Improving Blood Rheology and Microcirculation

Some studies have shown that the CPID patients are always under a situation of hypercoagulable blood. TCM defines this state as a blood stasis, and CHMs can accelerate blood circulation and eliminate stasis to improve this situation.

“Fuyanjingheji” (a kind of compound of CHM consisting of *Sparganium stoloniferum*, Rhizoma Curcuma, Semen Persicae, Fructus Meliae Toosendan, Rhizoma Alismatis, etc.) is used in CPID patients. A clinical research has shown that “Fuyanjingheji” could reduce the whole blood viscosity, blood plasma viscosity, and hematocrit, indicating that the drug can significantly improve the status of thickness, stickiness, and coagulation in blood of CPID patients [169]. “Penyanping,” an empirical Chinese herbal compound (consists of *Viola yedoensis* Makino, *Hedyotis diffusa*, *Forsythia suspensa*, *Parthenocissus himalayana planch*, *Salvia miltiorrhiza*, etc.), was used to treat CPID patients in Chinese clinic. Clinical research results indicated that “Penyanping” can improve the pelvic hemodynamics indexes, ovarian left arteriopalmus index, bilateral resistance index, maximal speed of left arterial blood flow, and score of time-velocity within CPID patients and prompt the absorption of inflammation [170]. “Manpenzhuyutang” is another Chinese herbal compound (consists of Pollen Typhae, Faeces Trogopterori, *Corydalis yanhusuo*, *Ligusticum chuanxion*, *Cyperus rotundus*, etc.) commonly used to treat CPID patients in Chinese clinic for a long period. One study proved it may improve that the blood rheology indicators in CPID rats model [171]. “Jingangteng Dispersible Tablet (JDT)” is a Chinese herbal patent used to treat CPID; the main composition of JDT is *Smilax*. An experiment indicated that JDT can promote blood circulation, dispel blood stasis, clear away pathogenic heat, and remove the toxin in CPID rat model. After treatment, the whole blood viscosity index significantly decreased, the state of blood stasis was improved, and the efficacy seemed to be dose dependent [172].

#### 2.2.5. The Mechanism of Chinese Herbal Medicine in Enhancing Immunity

The body's immune function is a key factor in reducing inflammation. A large number of experimental studies have shown that Chinese herbal compounds may improve the symptoms of immune disorders, thus reducing the inflammations of reproductive organs.

*(i) Enhancing the Function of Humoral Immunity*. As part of the humoral immune system, IgG, IgM, and IgA play a key role in protecting the body against infection [173]. Determining serum immunoglobulin (Ig) concentration may help estimate humoral immunity function. “Fuke Qianjin tablet,” a Chinese herbal patent, whose major ingredients are Philippine Flemingia Root, Radix Zanthoxyli, Root of Cherokee Rose, Andrographitis Paniculata, Radix Codonopsis, and so forth, was widely used to treat various gynecological inflammations. The experiment showed that “Fuke Qianjin tablet” was able to promote the production of IgA, IgG, and IgM in acute pelvic inflammatory disease (APID) rat model, thus improving the immune function and enhancing the antibacterial, anti-inflammatory, and anti-infection effects [174].

*(ii) Enhancing the Function of Cellular Immunity*. The ratio of CD4/CD8 was lower at the situation of chronic inflammation, which indicated the dysfunction of the immunity [175]. Additionally, TNF is an important cytokine to promote inflammation and has a good effect on the immune system at the initial stage of inflammation, but if it continues to rise, it would make a series of pathophysiological changes. One study showed that the ratios of CD4/CD8 and IL-2 were lower in women with CPID than non-CPID women, while the level of TNF-*α* was higher, indicating that CPID is closely related to the decline of immunity [176]. A clinical observation found that the ratios of CD4/CD8 and IL-2 were increased, while the level of TNF-*α* was decreased after treatment with “Penqiangyan granule” for CPID patients and rat model [177, 178]. Another Chinese herbal compound called “Tiedongqingtang” (consists of *Ilex purpurea* Hassk, Herba Taraxaci, *Carthamus tinctorius*, Angelica Sinensis, *Ligusticum chuanxiong*, Radix Paeoniae Rubra, etc.) was reported to improve the level of serum IL-2 in CPID patients, which might be one of the underlying mechanisms in enhancing immune function and improving the symptoms of CPID [179].

### 2.3. Acupuncture and Moxibustion for the Treatment of Genital Infection

As an important part of CAM, acupuncture and moxibustion, either used alone or in combination, can be an effective treatment for several diseases. The utilization of acupuncture is based on the meridian system: there are some acupoints along the meridian lines, which may have some beneficial effects on a certain disease when stimulated via needling, pressure, or heat [152]. Acupuncture directly acts on the lesion with a short treatment course and a reduced recurrence rate. Data mining of the literature revealed that acupoints on the Ren meridian were commonly selected to treat CPID. *Guanyuan* (CV4), *zigong* (EX-CA1), *zhongji* (RN3), *zusanli* (ST36), *sanyinjiao* (SP6), and *mingmen* (DU4) were the most frequently used for CPID [180]. *Ren* meridian is one of the eight extra meridians which originates in the lower abdomen. Acupuncture contains a variety of related treatment methods, such as moxibustion, auricular application, and pull cans. Acupuncture has achieved great success in treating gynecological inflammation of the reproductive system since it plays an important role in the anti-inflammatory processes. The mechanism may be due to activating the hypothalamic-pituitary-ovarian axis and the immune system, affecting the nerve-reproductive and endocrine-immune system, dilating blood vessels and lymphatic vessels, accelerating blood circulation, and inhibiting vascular permeability to reduce the inflammatory exudate and accelerate inflammatory exudate absorption [181].

#### 2.3.1. Acupuncture

Acupuncture is the most direct and basic method in this system. It requires using thin metal needles to pierce through skin into certain points to regulate the flow of qi around the whole body [182]. Acupoints are the special position where the “qi” of viscera and meridians intersect and effuse. Stimulating the acupoints can regulate “qi” and blood around the body. A special method of acupuncture puts an absorbable surgical suture (catgut) into the points as a kind of foreign protein. Sutures can therefore continuously stimulate acupoints, thereby allowing the body to produce some abnormal reactions to enhance the phagocytosis of white blood cells, which ultimately strengthens the anti-inflammatory effect.

#### 2.3.2. Acupoint Injection with Drugs

As one of the modern therapies, acupoint injection usually requires injecting a drug into acupuncture points to cure certain diseases. The drugs will be absorbed through the subcutaneous tissues and tiny blood vessels. The needles produce positive stimulation at local acupuncture points, which can promote the local blood circulation, elevate the metabolic ability, and ameliorate the pathology while promoting inflammation absorption [183]. Radix *Astragali* injection contains glycosides, polysaccharides, flavonoids, amino acids, and other chemical components, which has a significant effect on regulating immune function [184]. Clinical studies found that *Zusanli* (ST36) and *Guanyuan* (CV4) with Radix *Astragali* injections have better curative effect in treating CPID [185]. Secretory IgA (S-IgA) is the most important molecule secreted by the mucous in the humoral immune system, and the decreasing of S-IgA in local area is closely related to the increasing susceptibility to genial inflammation [186]. An experimental study showed that *Guanyuan* (CV4) and Zusanli (ST36) with Radix *Astragali* injection can increase the protein level of S-IgA in vaginal washing fluid of the CPID rat model, inhibiting excessive secretion of serum IL-6 and TNF-*α*. This had a certain effect on the abnormal expression of cytokines, which indicated that local immunity had increased after treatment [187].

#### 2.3.3. The Moxibustion

Moxibustion is a technique that produces heat by burning powdered herbal material at the acupoints. It works at the local skin by stimulating the skin receptors, affecting cellular metabolism, meeting the requirements of oxygen of the local tissue, and promoting the emissions of CO_2_ [188]. At the same time, moxibustion decreases the viscosity of the muscle fiber cells and accelerates blood circulation and eliminates the inflammation. Moxibustion is often combined with acupuncture and Chinese herbal medicine for treatment and has achieved satisfactory effects. One study made *Artemisia argyi*, Flos Carthami, Semen Persicae, *Paeonia lactiflora*, and Radix Aucklandiae into moxa sticks and placed them directly on the acupoints of *guanyuan* (CV4), *zhongji* (RN3), *zigong* (EX-CA1), *ciliao* (BL32), *sanyinjiao* (SP6), and *zusanli* (ST36) to treat CPID. All 38 cases had been cured with clinical symptoms disappearing after 5 periods of treatment, which achieved the desired effect [189].

#### 2.3.4. Acupoint Sticking

Acupoint sticking therapy is a process of making Chinese herbal medicine into different formulations such as pills, powders, and ointments and sticking it on the selected acupoints [190]. It can increase the local drug concentration and make the drug permeating directly on the lesion. Acupoint sticking can stimulate the pelvic nerve and promote blood flow to improve the absorption of inflammation. A clinical study used Herba Asari, Herba Ephedrae, *Semen sinapis* Rhizoma Corydalis, and so forth to treat CPID in the way of acupoint sticking and showed that the total effective rate was 93.33% [191].

#### 2.3.5. The Cupping Therapy

Cupping is a TCM therapy that dates back at least 2,000 years and has been applied as a formal modality in hospitals throughout China since 1950 [192]. In this method the negative pressure from cupping is one of the main factors that helps to achieve therapeutic effects. This method can improve the microcirculation and the blood rheology and increase the blood capillary permeability. Additionally, it may prompt inflammatory lesion absorption. Generally, negative pressure can extend local blood vessels to improve microcirculation, accelerate angiogenesis, and promote capillary endothelial cell repair [193]. One study treated 26 cases of CPID by the combination of acupuncture and cupping therapy. Results showed that after 6 months of therapy 20 cases had been cured and 6 cases improved [194].

#### 2.3.6. Others

In addition to the above treatments of acupuncture and moxibustion, there are some other related therapies including auricular therapy, pricking blood therapy, and burning acupuncture though they are not used widely in clinic. In addition, the method of the combination of acupuncture, moxibustion, and physical therapy is frequently used to specifically treat genital infection. One clinical study suggested that the total effective rate was 91.27% when treating 60 patients with PID via a combination of acupuncture, moxibustion, and ultrashort wave [195].

Acupoints are the most important elements in acupuncture prescription and choosing accurate and appropriate fixed acupoints in the clinic is directly related to the therapeutic effect of acupuncture. In addition, based on the fixed set of acupoints, we can increase or reduce acupoints according to the patient's clinical symptoms, which is also the most common method in clinical treatment. We identify commonly used acupoints for the treatment of PID in clinic (Table 4).

## 3. Physiotherapy in Genital Infection

In recent years, a series of clinical studies has explored the physical therapies used to treat female reproductive tract infections including electrotherapy, magnetic therapy, heat therapy, light therapy, and mud therapy. Physical therapy can be a physical factor used alone and can also be used in combination with two or more physical factors or with the use of drugs, which can enhance the efficacy and shorten the course of treatment. Here we review commonly used physical therapy treatments for gynecological diseases.

### 3.1. External High Frequency Calorimeter

Microwave hyperthermia can be used for the treatment of chronic pelvic inflammatory disease and vaginitis. With low frequency, long wavelength, and penetrating power, it is difficult to distribute the force generated inside to the rest of the body as almost all is absorbed as heat [196]. The mechanism includes improving the local tissue microcirculation, accelerating the elimination of the inflammatory substances accumulated in the local area, improving pelvic blood circulation and diseased tissue oxygen, increasing the metabolic rate, while reducing the excitability of sensory nerves and interfering with pain impulse conduction, and enhancing the ability of phagocytic cells, thus improving the body's immune function. Clinical studies demonstrated that enema combined with physiotherapy treatment for CPID has been successful and should therefore be adopted. In a clinical study examining physical therapy combined with traditional Chinese medicine retention enema to treat CPID, 32 cases were cured among 40 cases; in the observation group, the total efficiency reached 90% without any adverse reactions [197].

### 3.2. Low-Frequency Pulse Therapy

This type of therapy works by acting directly on local nerves and acting indirectly on body fluids, thus enhancing the therapeutic effect of CPID. Electromagnetic waves can adjust muscle contraction rate rising to the maximum point of physiological range and lead drug directly into the local lesion by affecting the magnetic field and maintaining a high local concentration, improving the effect of drug treatment [198].

### 3.3. Chinese Medicine Iontophoresis

Traditional Chinese medicine iontophoresis makes use of the principle that similar charges oppose each other and opposite charges attract in direct current (DC). In this way, Chinese medicine ions are able to penetrate into the corresponding lesions in the human body, to achieve the purpose of medical treatment [1]. In recent years, there have been numerous clinical reports about the effect of Chinese medicine iontophoresis treating CPID. In the electric field, Chinese medicine ions penetrate into the focus of infection, playing a dual therapeutic effect of DC and drug treatment [199]. Advantage of this method for CPID is that the drug works directly on the lesion area. The drug ions therefore generate a relatively higher concentration in the lesion area than that of oral drug which enters the blood circulation. Additionally, they have an extended duration, which extends the therapeutic effect at lower dosages compared to oral therapy and while achieving a comparable effect.

### 3.4. Ultrashort Wave Combined with Intermediate Frequency

Ultrashort wave in treating PID shows a good anti-inflammatory effect including (i) improving blood and lymph circulation, increasing vascular permeability, alkalinizing the pH of lesions, eliminating acidosis of local tissue, and promoting lesion organization drying to avoid the damaging effects of tissue edema. Further, it increases the ability of the reticuloendothelial system and white blood cells (WBC) and increases lectin and complement. This also helps to enhance the regeneration process of connective tissue, with good growth of granulation tissue, and thus localized inflammation and wound healing are enhanced. Ultrashort wave electric fields form a bad living environment for bacteria, indirectly inhibiting growth and reproduction of bacterium, and achieve the effect of sterilization, anti-inflammation, and analgesia to PID [200]. Computer intermediate frequency (IF) therapy apparatus is a kind of microcomputer-controlled and low-frequency modulated intermediate frequency current, which has a significant effect on improving local blood circulation, loosening adhesions, and softening the scar and analgesic [201]. Therefore, using ultrashort wave and computer IF in combination creates a synergistic effect, which can ultimately help eliminate pelvic inflammatory exudation and fibrosis. A clinical study demonstrated that the ultrashort wave combined computer IF group was significantly more effective compared to the simple ultrashort wave group [202].

### 3.5. Ozone Therapy

Clinical studies have shown that ozone treatment is a safe and effective method for female genital infection, especially for vaginal inflammation. Ozone possesses efficient, rapid, and broad-spectrum antiseptic qualities and plays a key role in killing pathogenic microorganisms. Gynecological ozone instrument is based on the technique of low temperature plasma which produces high concentrations of ozone by ionizing the gas molecules in the air. The instrument consists of two parts: one part homogenizes the ozone concentration of an entire portion of the reproductive tract and kills a variety of pathogenic microorganisms in doing so; the other part of the fumigation is to take advantage of the superior bactericidal capacity of ozone, in order to make ozone disperse directly into the area and complete sterilization. Ozone liquid will not damage the acid-base balance in the vagina and reverts into pure oxygen rapidly, which will allow the normal growth of vaginal bacteria and inhibit the growth of anaerobic bacteria [203].

## 4. Psychological Intervention Therapy

Many women who suffer from genital infection are at risk of psychological problems including anxiety and depression. Related studies show that psychosocial factors play an important role in the occurrence and development of the disease [204]. Patients with genital inflammation fear repeated course and bear heavy psychological burden for a long time, which may lead to depression, irritability, and anxiety, thereby aggravating the disease. The appropriate psychotherapy may help achieve beneficial results in treating genital infection.

### 4.1. Support

Expressing concern and understanding for the patient's suffering, explaining each of the issues raised by patients, establishing a good doctor-patient relationship, and obtaining the trust and cooperation from the patients are key to maintaining adequate psychosocial health for women who may be experiencing genital infection.

### 4.2. Acknowledge

Conducting depression, anxiety, and disease-related lectures—such as teaching patients about the pathogenesis, clinical manifestations, complications, and treatment of the disease—can help educate patients making them mentally confident in overcoming it.

### 4.3. A Combined Therapy of TCM and Psychological Intervention

Patients who suffer from genital infection are always under the situations of anxiety and depression. Psychological treatment could enhance resilience and positive affect, then improving health and well-being, so psychological treatment combined with TCM not only cures the disease itself but also could provide additional efficacy of improving the quality of life by regulating the psychological situations for those patients undergoing genital infection.

A clinical study indicated that psychological treatment united with Chinese herbal patent was significantly better than traditional therapies for chronic pelvic pain [205]. *“Xiaozhengsan”* is a traditional Chinese herbal compound (consists of Rhizoma Homalomenae, *Zanthoxylum piperitum* DC, Cortex Acanthopanacis, Radix Angelicae Dahuricae, *Taxillus chinensis*, Radix Paeoniae Rubra, etc.) that has been used in treating gynecologic diseases for years; applying *Xiaozhengsan* externally combined with psychological and behavioral intervention could significantly improve the efficacy and patients' physiological and psychological quality of life [206]. Simple select 164 patients with chronic pelvic inflammatory disease were divided into two groups; the results showed that psychological care stress therapy combined with enema and DC therapy had a significantly greater effect over the control group without psychological care [207].

### 4.4. Music Therapy

Music care and alternative therapies in clinical applications are becoming increasingly widespread [208]. One study showed that 180 women with pelvic inflammatory disease were randomly divided into a treatment group and a control group. Women in the control group were treated with anti-CPID drugs and pelvic therapy instruments, while women in the treatment group were treated with the same drugs, pelvic therapy instrument, and music therapy. The anxiety scores in the music group significantly decreased when compared to the control group after treatment, and the total average time to improve efficiency and symptoms were significantly better than the control group. Music therapy is a common method of psychological intervention, which has been widely used in the treatment and care of patients in clinical practice [209, 210]. The majority of patients suffer from anxiety, insomnia, and lack of energy, which seriously affect the rehabilitation of the disease. Music affects the brain in the form of sound waves and stimulates the pituitary gland to release morphine-like substances, which can inhibit pain impulses [211]. As a special psychological treatment, music therapy can improve anxiety, relieve pain, and increase comfort among susceptible patients. Additionally, it is more economical and an easier lifestyle modification for patients to attempt and accept.

## 5. Medical Rehabilitation 

Medical rehabilitation gymnastics is a very important content for CPID patients in their remission stage, because it helps regulate the function of the autonomic nervous system, relieve local pain, improve metabolism, and relieve pelvic inflammatory adhesions, thus improving the quality of life of patients with CPID. Studies show that Kung-Fu support is playing a positive role in improving the quality of patients' lives. After accepting pelvic medical rehabilitation treatment for 3 months, scores of each index of CPID patients had increased [212, 213], which indicated an improved quality of life score.

## 6. Conclusion and Future Directions

Genital infection is a widespread gynecologic disease, particularly among reproductive age women. If inflammation is left untreated, it will negatively impact the women's immune system, metabolism, and endocrine system. There are additional considerations with pregnant women such as causing intrauterine infection and reproductive tract and neonatal infection, both leading to serious consequences. Numerous clinical studies in vitro and in vivo have shown that TCM may be an effective treatment for genital infection and will not cause tolerance and adverse reactions after prolonged use. Chinese medicines treat genital infection based on the holistic concept and the theory of syndrome differentiation treatment. TCM has achieved the clinical curative effect of antibacterial and anti-inflammatory therapeutics, thus improving both immunity and microcirculation. Chinese medicine has made remarkable achievements in the treatment of genital infection, and this paper reviews select effective Chinese herbal compounds that are commonly used. Chinese medicine treatment works through a variety of mechanisms: oral administration, external application, and acupuncture. Whether used as single or incombination therapy can affect how patients may respond to various types of treatment regimens. However, it would be beneficial to conduct large-scale, randomized clinical trials in the future to identify the efficacy of CAM and help substantiate its therapeutic effect.

## Figures and Tables

**Table 1:** List of effective Chinese herbal compounds for genital infection with oral administration.

The frequency of related articles	Chinese herbal compound	Formula composition	Disease	The average effective rate
*N* = 41.38%* *[4–15]	Shaofuzhuyutang	*Foeniculum vulgare*, Rhizoma Zingiberis, Rhizoma Corydalis, *Commiphora molmol*, Radix Angelicae Sinensis, *Ligusticum chuanxiong*, Cortex Cinnamomi, Radix Paeoniae Rubra, Pollen Typhae, Faeces Togopterori	PID	RR, 1.23 (1.17, 1.28)

*N* = 10.34%* *[16–18]	Dangguishaoyaosan	Radix Angelicae Sinensis, Chinese herbaceous peony, *Poria*, *Atractylodes macrocephala*, Rhizoma Alismatis, *Ligusticum chuanxiong*	PID	RR, 1.44 (1.22, 1.71)

*N* = 13.79%* *[19–22]	Guizhifulingtang	Ramulus Cinnamomi, *Poria*, *Glycyrrhiza uralensis*, Cortex Moutan, Chinese herbaceous peony, Semen Persicae	PID	RR, 1.24 (1.14, 1.35)

*N* = 10.34%* *[23–25]	Danzhixiaoyaosan	*Atractylodes macrocephala*, Radix Bupleuri, Radix Angelicae Sinensis, *Poria*, *Glycyrrhiza uralensis*, Cortex Moutan, *Gardenia jasminoides*, Chinese herbaceous peony	PID	RR, 1.22 (1.11, 1.34)

*N* = 17.24%* *[26–30]	Bixieshenshitang	Rhizoma Dioscoreae Collettii, Semen Coicis, Rhizoma Smilacis Glabrae, Pulvis Talci, Cortex Moutan, Rhizoma Alismatis, Medulla Tetrapanacis, Cortex Phellodendri	Vaginitis	RR, 1.14 (1.07, 1.22)

*N* = 6.90%* *[31, 32]	Wandaitang	*Atractylodes macrocephala*, Rhizoma Dioscoreae, Ginseng, Chinese herbaceous peony, Semen Plantaginis, Rhizoma Atractylodis, *Glycyrrhiza uralensis*, Pericarpium Citri Reticulatae, *Schizonepeta tenuifolia *	Cervicitis/vaginitis	RR, 1.37 (1.22, 1.54)

**Table 2:** List of effective Chinese herbal compounds for genital infection with external therapy of TCM.

The frequency of related articles	Chinese herbal compound	Formula composition	Administration route	Disease	The average effective rate
*N* = 47.37% [34–42]	Hongtengtang	Caulis Sargentodoxae, Herba Patriniae, Herba Taraxaci, Rhizoma Sparganii, Rhizoma Curcumae, Radix Astragali	Rectal	PID	RR, 1.31 (1.23, 1.39)

*N* = 10.53% [43, 44]	Kushentang	Radix Sophorae Flavescentis, Fructus Cnidii, Radix Angelicae Dahuricae, Rhizoma Atractylodis, Fructus Kochiae, Cortex Phellodendri	Vaginal lavage/steaming washing/intravaginal administration	Vaginitis/cervicitis	RR, 1.33 (1.18, 1.51)

*N* = 15.79% [45–47]	Shechuangzisan	Fructus Cnidii, Fructus Kochiae, Radix Sophorae Flavescentis, *Stemona sessilifolia*, Cortex Phellodendri Chinensis, Cortex Dictamni	Steaming washing/vaginal lavage	Vaginitis	RR, 1.15 (1.08, 1.24)

*N* = 26.32% [33, 48–51]	Longdanxiegantang	Radix Gentianae, Radix Scutellariae, *Gardenia jasminoides* Ellis, Rhizoma Alismatis, Caulis Akebiae, Semen Plantaginis, *Angelica sinensis*, Radix Rehmanniae, Radix Bupleuri, *Glycyrrhiza uralensis *	Steaming washing	Vaginitis	RR, 1.07 (1.03, 1.12)

**Table 3:** Frequent Chinese herbal medicine in internal and external therapies.

	PID	Frequency [65–81]	Vaginitis	Frequency [82–93]	Cervicitis	Frequency [94–99]
Frequent Chinese herbal medicine in oral administration of CHM	Radix Paeoniae Rubra	*N* = 14	Rhizoma Atractylodis Macrocephalae	*N* = 10	Rhizoma Atractylodis Macrocephalae	*N* = 11
	* Sargentodoxa cuneata*	*N* = 13	Dioscorea Opposita	*N* = 10	Root of Chinese Thorowax	*N* = 6
	Cortex Moutan Radicis	*N* = 11	*Plantago asiatica* L.	*N* = 8	Dioscorea Opposita	*N* = 5
	* Salvia miltiorrhiza*	*N* = 11	*Wolfiporia cocos *	*N* = 7	*Wolfiporia cocos *	*N* = 5
	* Ixeris denticulata*	*N* = 11	Root of Chinese Thorowax	*N* = 7	White Pieony Root	*N* = 5
	Herba Taraxaci	*N* = 9	Cortex Phellodendri Chinensis	*N* = 6	Radix Codonopsis Pilosulae	*N* = 4
	*Wolfiporia cocos *	*N* = 9	Radix Codonopsis Pilosulae	*N* = 6	Rhizoma Atractylodis	*N* = 4
	Radix Angelicae Sinensis	*N* = 9	Pericarpium Citri Reticulatae	*N* = 5	Radix Angelicae Sinensis	*N* = 4

	PID	Frequency [100–116]	Vaginitis	Frequency [117–132]	Cervicitis	Frequency [133–148]

Frequent Chinese herbal medicine in external therapy	*Ixeris denticulate*	*N* = 14	Radix Sophorae Flavescentis	*N* = 15	Cortex Phellodendri Chinensis	*N* = 11
	Semen Persicae	*N* = 9	Fructus Cnidii	*N* = 14	Borneol	*N* = 10
	Herba Taraxaci	*N* = 9	Cortex Phellodendri Chinensis	*N* = 12	Radix Sophorae Flavescentis	*N* = 10
	Radix Paeoniae Rubra	*N* = 8	*Stemona sessilifolia *	*N* = 11	Fructus Cnidii	*N* = 7
	*Sargentodoxa cuneata*	*N* = 8	Cortex Dictamni	*N* = 9	*Coptis chinensis* Franch	*N* = 7
	Rhizoma Sparganii	*N* = 8	Fructus Kochiae	*N* = 7	Borax	*N* = 3

**Table 4:** The list of available acupoints and the frequency of them in treating PID.

	Acupuncture point	English name	The frequency of related points Totle: 107
The main points	Sanyinjiao	SP6	*N* = 40
	Guanyuan	CV4	*N* = 38
	Zhongji	RN3	*N* = 36
	Zusanli	ST36	*N* = 28
	Qihai	CV6	*N* = 25
	Zigong	EX-CA1	*N* = 23
	Guilai	ST29	*N* = 20

The minor points	Shenshu	BL23	*N* = 15
	Ciliao	BL32	*N* = 15
	Xuehai	SP10	*N* = 15
	Yinlingquan	SP9	*N* = 14
	Shuidao	ST28	*N* = 10
	Taichong	LR3	*N* = 4
	Diji	SP8	*N* = 4
	Daimai	GB26	*N* = 4
	Daheng	SP15	*N* = 3
	Qichong	ST30	*N* = 3
	Baihuanshu	BL30	*N* = 3
	Mingmen	DU4	*N* = 3
